# Solitary extramedullary plasmacytoma of the tongue: a case report

**DOI:** 10.1097/RC9.0000000000000402

**Published:** 2026-03-18

**Authors:** Mohamed Bouallou, Achraf Amine Sbai, Drissia Benfadil, Azeddine Lachkar, Fahd El Ayoubi El Idrissi

**Affiliations:** a Faculty of Medicine and Pharmacy, Mohammed First University, Oujda, Morocco; b Department of Otorhinolaryngology, Mohammed VI University Hospital, Oujda, Morocco; c Mohammed First University, LAMCESM, Oujda, Morocco; d Laboratory of Oto-Neuro-Ophthalmology (LORNO), Faculty of Medicine and Pharmacy, Mohammed First University, Oujda, Morocco

**Keywords:** case report, extramedullary plasmacytoma, plasma cell dyscrasia, radiotherapy, tongue

## Abstract

**Introduction and Importance:**

Extramedullary plasmacytomas are rare solitary plasma cell neoplasms that occur outside the bone marrow. Primary tongue involvement is exceptionally uncommon and presents a significant diagnostic challenge. Their clinical presentation is often subtle and may closely mimic more common neoplastic processes.

**Case Presentation:**

We report the case of a 35-year-old man who presented with a gradually enlarging, firm, and painless mass on the ventral surface of the tongue that had been evolving for 5 months. Histopathological and immunohistochemical analyses revealed sheets of atypical plasma cells expressing CD38 and CD138 with kappa light-chain restriction, consistent with monoclonal plasma cell proliferation. A comprehensive systemic evaluation excluded multiple myeloma. The patient underwent definitive radiotherapy with a total dose of 50 Gy delivered in 25 fractions, resulting in complete remission without any functional impairment. At 9 months of follow-up, the patient showed no evidence of local recurrence or progression to multiple myeloma.

**Clinical Discussion:**

Primary extramedullary plasmacytoma of the tongue is exceptionally rare and may clinically mimic more common malignant or benign lesions, particularly in patients with a history of tobacco and alcohol exposure. An accurate diagnosis requires a multidisciplinary approach that integrates clinical findings, imaging, and histopathology, with immunohistochemical confirmation of monoclonality. Currently, radiotherapy remains the cornerstone of treatment, providing excellent local control and durable long-term remission.

**Conclusion:**

This case underscores the exceptional rarity of primary extramedullary plasmacytoma of the tongue and highlights the effectiveness of radiotherapy as a curative and function-sparing treatment option for this condition, while emphasizing the necessity of long-term and lifelong surveillance to detect potential late recurrence or progression to multiple myeloma.

## Introduction

Plasma cell neoplasms constitute a heterogeneous group of disorders arising from terminally differentiated B lymphocytes and encompass multiple myeloma, solitary plasmacytoma of the bone (SPB), and extramedullary plasmacytoma (EMP)^[^1^]^. EMP is an uncommon malignant proliferation of monoclonal plasma cells that form a solitary mass in soft tissues outside the bone marrow. It occurs in the absence of significant marrow infiltration (<10%), systemic multiple myeloma, or myeloma-related end-organ damage^[^2^]^. Approximately 40% of EMP cases eventually progress to multiple myeloma^[^3^]^. Solitary EMP accounts for 5 % of all plasma cell malignancies and 1%–2% of all head and neck tumors^[^4^]^. Primary EMP of the tongue is exceedingly uncommon, particularly in young adults, as EMP typically affects individuals between 50 and 70 years of age^[^5^]^. Tongue involvement is exceptionally rare, making diagnosis and management particularly challenging.

HIGHLIGHTS

- Extremely rare case: This report describes a primary extramedullary plasmacytoma of the tongue, an unusual site that may easily be misdiagnosed.
- Diagnostic insight: Highlights the importance of correlating clinical, imaging, and histopathological findings for the accurate identification of tongue lesions.
- Successful conservative therapy: Radiotherapy can achieve complete remission while preserving tongue function, offering a safe and effective treatment strategy.

Its clinical presentation may resemble that of more common malignant lesions of the oral cavity, particularly in patients with chronic tobacco exposure, which may contribute to diagnostic delays. The diagnosis of EMP relies on histopathological confirmation of monoclonal plasma cell infiltration, supported by immunohistochemistry, and on the exclusion of systemic multiple myeloma through comprehensive clinical, biological, and radiological staging. Early stage EMP generally has a favorable prognosis, especially when it arises in the head and neck region^[^6^]^. In this context, our case underscores the excellent therapeutic response to radiotherapy, further supporting its role as the standard treatment for localized EMP.

In this report, we describe a rare primary EMP of the tongue in a young adult, highlighting its diagnostic challenges and excellent therapeutic response to radiotherapy. This case report has been reported in line with the SCARE 2025 guidelines^[^7^]^.

## Case presentation

A 35-year-old Moroccan man with a medical history of a right forearm fracture managed with osteosynthesis 3 months earlier and a longstanding history of tobacco and alcohol use presented with a progressively enlarging mass of the tongue. The lesion, located on the left ventral surface of the mobile tongue, gradually increased in size over the preceding 5 months.

### Timeline of events

**Day 1:** Onset of a progressively enlarging mass on the left ventral surface of the tongue.

**Day 87**: Consultation with a general practitioner; treated with a povidone-iodine mouthwash.

**Day 145**: Presentation to the Otorhinolaryngology Department for evaluation.

**Day 147**: Biopsy of the lesion confirmed an EMP of the tongue.

**Day 159**: Completion of baseline laboratory investigations.

**Day 163**: Cervico-thoraco-abdomino-pelvic CT scan showing no evidence of secondary localization of the tumor.

**Day 169**: Multidisciplinary team discussion led to the decision to proceed with definitive radiotherapy.

**Day 175**: Initiation of radiotherapy with a total dose of 50 Gy delivered in 25 fractions over 5 weeks.

**Day 213**: Completion of the radiotherapy.

**Day 291**: Complete regression of the lesion with no radiotherapy-related adverse effects and full preservation of mastication.

On admission, the patient was alert, oriented, and afebrile (37.1°C). The patient was hemodynamically stable (blood pressure, 133/77 mmHg) with normal respiratory function (oxygen saturation, 99%).

**Figure 1.**
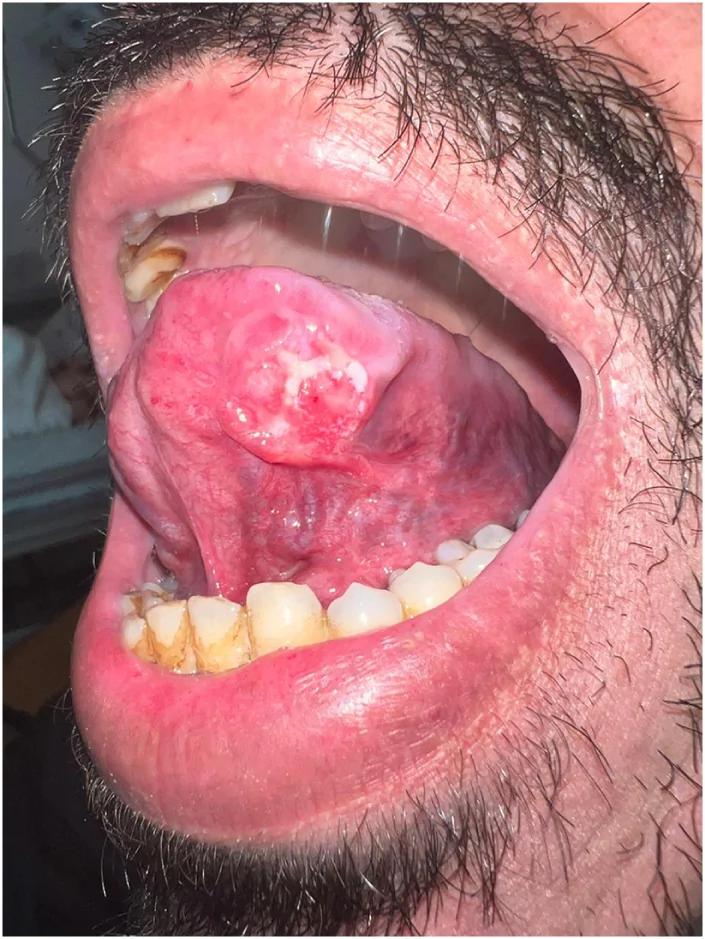
Clinical image demonstrating an oval, well-defined yet infiltrative mass on the ventral aspect of the left hemilingual surface.

Physical examination revealed poor oral hygiene and an ill-defined, infiltrative, well-circumscribed, oval mass on the ventral aspect of the left hemilingual surface, abutting the lateral free edge of the tongue (Fig. 1). The lesion measured approximately 3 cm in its greatest dimension. On palpation, the mass was firm and indurated, non-tender, non-hemorrhagic on contact and showed no extension to the floor of the mouth or crossing the midline. Cervical examination revealed no palpable lymphadenopathy, and flexible nasofibroscopy revealed no abnormalities of the upper aerodigestive tract.

**Figure 2.**
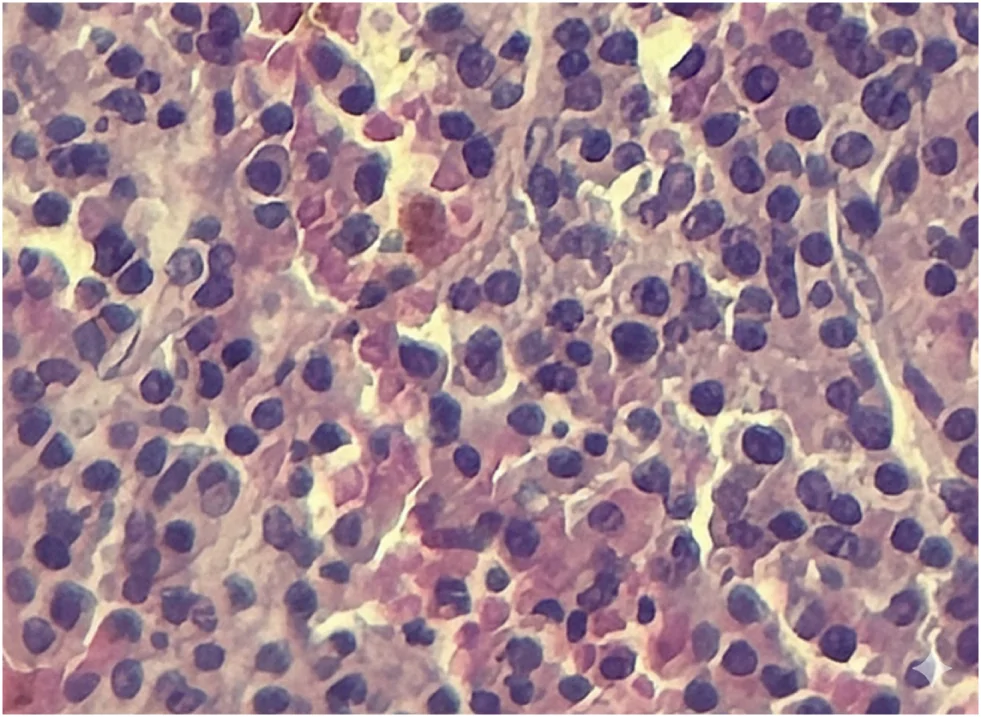
Histopathological section showing diffuse proliferation of plasma cells with eccentric nuclei and abundant eosinophilic cytoplasm, consistent with plasmacytoma.

Under local anesthesia, two incisional biopsies of the lesion were performed by a senior otorhinolaryngology professor with more than 30 years of clinical experience. Histopathological examination revealed diffuse tumor proliferation composed of plasma cells with eccentric nuclei and abundant eosinophilic cytoplasm (Fig. 2). Immunohistochemical analysis revealed strong positivity for CD38 and CD138, with kappa light-chain restriction, while CD19, CD20, HHV8, and cyclin D1 were negative.

Given the patient’s longstanding history of alcohol and tobacco use, the initial differential diagnoses included squamous cell carcinoma; by far the most common oral cavity malignancy in smokers; as well as inflammatory pseudotumor, lymphoma, Kaposi sarcoma, and Langerhans cell histiocytosis.

A comprehensive systemic workup was performed to rule out multiple myeloma. Contrast-enhanced computed tomography of the head, neck, thorax, abdomen, and pelvis revealed no evidence of nodal involvement, osseous destruction, or distant metastasis. MRI of the tongue could not be performed due to the presence of ferromagnetic osteosynthesis material. Laboratory investigations revealed normal blood counts, calcium levels, renal function, albumin levels, uric acid levels, and total protein levels. Lactate dehydrogenase and erythrocyte sedimentation rates were within normal limits. Serum protein electrophoresis with immunofixation showed no evidence of monoclonal (M) protein, and Bence–Jones proteinuria was negative. Bone marrow aspiration results were normal.

After a multidisciplinary discussion with the oncology department, a diagnosis of primary solitary EMP of the tongue was established in accordance with the International Myeloma Working Group diagnostic criteria.

**Figure 3.**
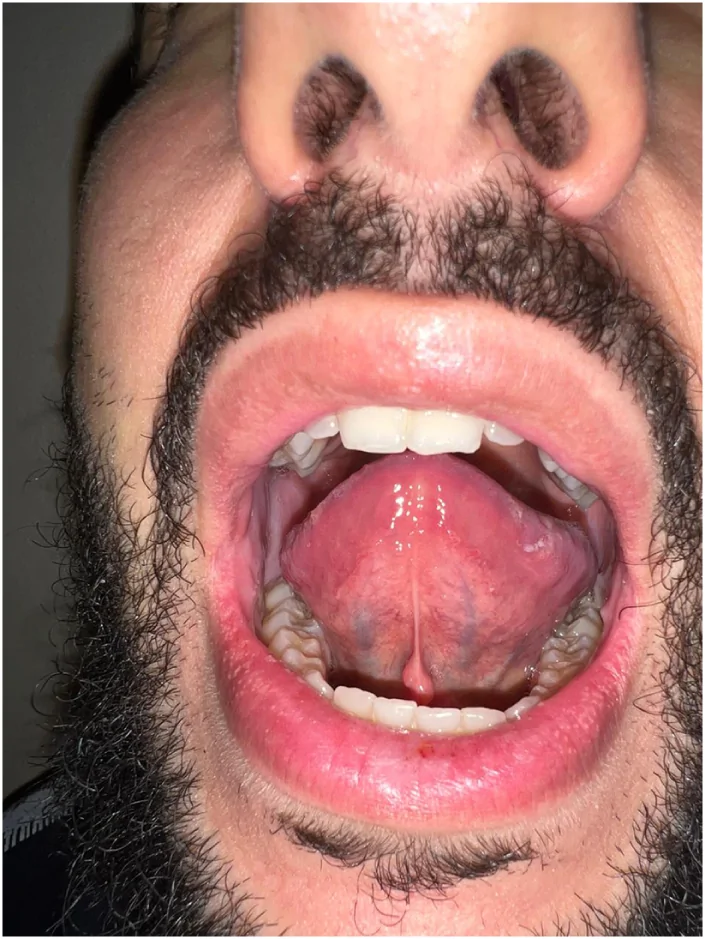
Follow-up clinical photograph demonstrating full resolution of the lesion at 9 months after radiotherapy.

The patient received definitive radiotherapy to the lesion, with a total dose of 50 Gy delivered in 25 fractions over 5 weeks. The treatment was well tolerated, with no reports of significant mucositis or xerostomia. At the 9-month follow-up, the patient was reassessed in an outpatient consultation, where clinical and radiological evaluations confirmed complete remission with no evidence of local recurrence or systemic progression (Fig. 3). The patient expressed appreciation for the rapid diagnostic clarification and non-invasive nature of radiotherapy.

The patient reported complete resolution of symptoms and expressed high satisfaction with the treatment outcome, with preserved tongue mobility and normal speech and swallowing function.

## Discussion

EMP of the tongue is exceedingly rare, with only a limited number of cases reported in the literature, accounting for approximately 1% of all head and neck tumors^[^8^]^. Compared to solitary bone plasmacytoma (SBP), solitary EMP is considerably less common. This rarity, combined with the potential for misdiagnosis as more prevalent malignant or inflammatory lesions, underscores the diagnostic challenge and clinical relevance of this case.

Plasmacytomas are classically divided into three entities: multiple myeloma, SBP, and EMP. While EMPs represent a small proportion of plasma cell neoplasms, they demonstrate a strong predilection for the upper aerodigestive tract, with nearly 80% of cases arising in this region, including the oral cavity^[^9^]^. Our patient was 35 years old, which is an unusually young age for this condition, thereby expanding the known age spectrum and illustrating that EMP should remain in the differential diagnosis of tongue masses even in younger adults.

Clinically, EMP of the oral cavity typically present as firm, well-circumscribed, submucosal nodules that remain asymptomatic until progressive enlargement causes discomfort, dysarthria or dysphagia^[^10^]^. In our case, the lesion was localized to the left ventral surface of the mobile tongue, without ulceration, pain, or cervical lymphadenopathy, which may mimic benign conditions or early-stage squamous cell carcinoma, particularly in the context of active tobacco use. This emphasizes the necessity of histopathological confirmation for accurate diagnosis. This clinical overlap reinforces the importance of histopathological confirmation to establish an accurate diagnosis, especially in cases of lesions refractory to symptomatic treatment for 15 days.

In this clinical context, the differential diagnosis of a tongue mass is particularly broad and encompasses a wide spectrum of benign, inflammatory, infectious, and malignant conditions. Among adults, squamous cell carcinoma represents the most common malignant neoplasm of the oral cavity, particularly in patients with chronic tobacco and alcohol exposure, and thus constitutes the primary diagnostic consideration^[^11^]^. Its early clinical presentation can closely mimic that of EMP, especially in the absence of ulceration or cervical lymphadenopathy. Other malignant entities, including non-Hodgkin lymphoma, Kaposi sarcoma in immunocompromised individuals, and salivary gland–derived tumors such as mucoepidermoid and adenoid cystic carcinomas, should also be considered.

Furthermore, several benign conditions, notably granular cell tumors, inflammatory pseudotumors, vascular malformations, and Langerhans cell histiocytosis, may further complicate the diagnostic assessment due to their similar submucosal appearance. Given this substantial overlap in presentation, histopathological evaluation with immunohistochemical confirmation of monoclonal plasma cell proliferation, along with exclusion of systemic involvement, is essential. This highlights the critical importance of early biopsy and a multidisciplinary approach in the management of persistent or atypical lingual lesions

The diagnosis of EMP mandates a comprehensive multidisciplinary approach combining clinical, histopathological, immunohistochemical, and systemic evaluations. Histologically, EMP is characterized by sheets of mature and atypical plasma cells with eccentric nuclei and abundant eosinophilic cytoplasm, typically expressing CD138 and/or CD38^[^12^]^. Confirmation of monoclonality is essential and can be achieved by demonstrating light-chain restriction (kappa or lambda) on immunohistochemistry or through polymerase chain reaction–based studies. In the present case, the tumor cells showed strong CD38 and CD138 positivity with kappa light-chain restriction, findings consistent with clonal plasma cell proliferation.

The exclusion of systemic diseases is paramount. According to the International Myeloma Working Group (IMWG) criteria, EMP is defined by: (1) a solitary extramedullary lesion, (2) absence of bone marrow plasmacytosis exceeding 10%, (3) no osteolytic lesions on skeletal imaging, and (4) absence of serum or urinary monoclonal components suggestive of systemic myeloma^[^12^]^. All these criteria were satisfied in our patient, as confirmed by normal bone marrow aspiration, absence of M-protein, and negative Bence–Jones proteinuria results.

While the exact etiology of EMP remains incompletely understood, these tumors are thought to arise from post–germinal center B cells that undergo neoplastic transformation under the influence of chronic antigenic stimulation and cytokines, such as interleukin-6^[^13^]^. Cytogenetic abnormalities, particularly 13q deletions and structural rearrangements involving 14q, have been described in plasmacytic neoplasms and may contribute to clonal evolution^[^14^]^. These molecular alterations likely interact with the local inflammatory microenvironment to promote plasma cell proliferation and survival.

Despite their locally invasive and potentially aggressive biological behavior, radiotherapy remains the cornerstone of treatment for solitary EMP due to the pronounced radiosensitivity of plasma cells. In our patient, a total dose of 50 Gy delivered in 25 fractions achieved complete remission without any functional or aesthetic sequelae. This therapeutic strategy is consistent with the findings of Creach *et al*, who reported local control rates exceeding 85% in head and neck EMPs treated with definitive radiotherapy at approximately 50 Gy, with minimal toxicity and durable remission^[^15^]^. Similarly, Oertel *et al* observed that radiation doses ≥ 45 Gy were associated with improved local control and overall survival, further highlighting the radiosensitivity of plasma cell neoplasms and reinforcing radiotherapy as an effective single-modality treatment for localized disease^[^16^]^.

Surgical resection may be considered in selected cases with small, well-demarcated lesions or for diagnostic confirmation purposes. However, in anatomically and functionally critical sites, such as the tongue, definitive radiotherapy offers excellent local control while preserving speech, swallowing, and mobility. Notably, a recent systematic review and meta-analysis demonstrated that radiotherapy was associated with superior 5-year disease-free survival compared with surgery alone^[^17^]^. By contrast, the role of adjuvant chemotherapy remains controversial and is generally reserved for cases of recurrence, residual disease, or progression to multiple myeloma.

EMP usually follows an indolent course compared to bone plasmacytomas^[^18^]^. Nonetheless, progression to multiple myeloma occurs in approximately 10% of cases, typically within the first 3 years, with the risk of progression being markedly higher in cases of SBP compared to EMP^[^12^]^. Prognostic factors influencing this evolution include tumor size (> 5 cm), incomplete local control, and persistence of serum monoclonal protein after treatment^[^19^]^. Our patient demonstrated none of these adverse indicators and remained in complete remission, which correlates with a favorable prognosis.

Structured and prolonged follow-up is essential. Periodic evaluation with physical examination, serum protein electrophoresis, and imaging is required for the early detection of recurrence or progression to multiple myeloma. Given the possibility of late relapse despite initial complete remission, lifelong surveillance is strongly recommended^[^12^]^.

This case underscores key clinical lessons for the evaluation of tongue masses. EMP should be considered even in younger adults, particularly when a lesion persists beyond 15 days despite appropriate treatment. Given the nonspecific clinical presentation, an early biopsy is essential. The case further highlights the importance of a structured, multidisciplinary diagnostic workup to accurately differentiate localized EMP from other potential differential diagnoses. Finally, the excellent therapeutic response observed supports radiotherapy as an effective, function-preserving treatment modality for tongue lesions. Collectively, these findings emphasize the need for early recognition and individualized management to optimize patient outcomes.

## Conclusion

EMP of the tongue is an exceptionally rare diagnosis that should be considered even in younger patients with tongue masses. Histopathological and immunohistochemical evaluations are essential for accurate diagnosis. Definitive radiotherapy remains the treatment of choice, providing excellent local control and functional preservation. Lifelong clinical and biological surveillance is recommended because of the potential for late recurrence or progression to multiple myeloma.

## Data Availability

The dataset s supporting the findings of this report are stored in the institutional ENT database and are available from the corresponding author upon reasonable request.
